# *Cyclospora* infection in a young woman with human immunodeficiency virus in Hong Kong: a case report

**DOI:** 10.1186/1756-0500-6-521

**Published:** 2013-12-09

**Authors:** Owen Tak-yin Tsang, Richard Wing-cheuk Wong, Bosco Hoi-shiu Lam, Jacky Man-chun Chan, Kay-yan Tsang, Wai-shing Leung

**Affiliations:** 1 Hospital Authority Infectious Disease Centre at Princess Margaret Hospital, 2-10 Princess Rm 313, Blk S, Margaret Hospital Rd, Lai Chi Kok, Hong Kong, SAR, China; 2 Department of Pathology, Princess Margaret Hospital, Hong Kong, China

**Keywords:** *Cyclospora*, Protozoa, HIV, Prolonged diarrhoea

## Abstract

**Background:**

*Cyclospora* is an uncommon pathogen. The diagnosis of *Cyclospora* infection can be difficult because of its scarcity in developed countries, intracellular mode of life, small size of the parasite and its inability to take up routine microscopic stains. However, it is endemic in many countries in Asia, Africa, Central and South America. With the increase in travels to these areas, the number of cases is expected to increase. Moreover, it is found to be associated with numerous food-borne outbreaks.

**Case presentation:**

We encountered a patient with human immunodeficiency virus presented with 6 months of diarrhoea. The initial investigation was unrevealing. The diagnosis of *Cyclospora* infection was finally made on the histological sample obtained by colonoscopy. Moreover, the initial therapy with ciprofloxacin was not effective, while trimethoprim/sulfamethoxazole resulted in final cure of the disease.

**Conclusion:**

Travel and food histories are important for the suspicion of *Cyclospora* infection. Histological examination is more sensitive in making a diagnosis of *Cyclospora* infection of the gut than fecal microscopic examination. Trimethoprim/sulfamethoxazole is a more reliable therapy for *Cyclospora* infection in patients with human immunodeficiency virus.

## Background

*Cyclospora* is a rare pathogen even in human immunodeficiency virus (HIV)-infected population. *Cyclospora* species belong to the subphylum Apicomplexa, subclass Coccidiasina, and family Eimeriidae. It was first described to be a coccidian parasite causing diarrhoea in patients of Papua New Guinea in 1979 by Ashford though it was thought to be the parasite of the genus *Isospora* at that time [1]*.* The parasite was finally named *Cyclospora cayetanensis* when it was noted to be a distinct organism by morphological and serological examinations [2]. *C. cayetanensis* has been identified only in humans to date [3]. It is endemic in many regions including India, Indonesia, Thailand, some parts of Africa, Central and South America [4]. It is a significant culprit for travelers’ diarrhoea in developed countries and has been found to be associated with numerous food-borne outbreaks. Food items typically noted to be associated with the infection are fresh fruits and vegetables like raspberries, blackberries, basil and lettuce [4]. The prevalence in developed countries like the United States can vary from 0.01 cases per 100,000 persons to 0.07 cases [5]. In endemic areas, the prevalence is found to be high in children. In a 2-year cross-sectional study of 5836 Peruvian children, the overall prevalence of *Cyclospora* infection by examining their fecal samples for oocysts, was 1.1% with a peak in those aged 2 to 4 years old (2%) [6]. Another possible risk factor for *Cyclospora* infestation is HIV infection. An Indian case–control study demonstrated that the fecal positive rate of *Cyclospora* in HIV positive patients is much higher than that of the HIV negative control (20.44% vs 1.5%, respectively) [7]. We present here an HIV patient with *Cyclospora* infection and highlight some of the difficulties in the diagnosis and treatment of this disease.

## Case presentation

A 38-year-old lady who was otherwise healthy presented with 6-month history of intermittent diarrhoea since her trip to Vietnam 2 weeks before the onset of symptoms. She had no fever, vomiting or abdominal pain. Her stool was watery with no blood or mucus. Her symptoms did not subside despite treatment prescribed by general practitioners. She also had severe weight loss of 5 kg over 6 months. She claimed that she had taken some homemade salad during her stay in Vietnam for her holiday. She worked as a dental nurse in Hong Kong while her husband stayed in Vietnam and used to visit her twice yearly. She did not take any long-term medications before the onset of symptoms nor have any sign and symptoms suggestive of hyperthyroidism. Her blood counts including hemoglobin, white blood cells and platelet counts were normal when she presented to us 6 months after onset of symptoms. The renal and liver functions tests were also unremarkable expect for a low albumin level of 33 g/L. Her thyroid function tests, fasting glucose, calcium and phosphate levels were all normal.

**Figure 1:**
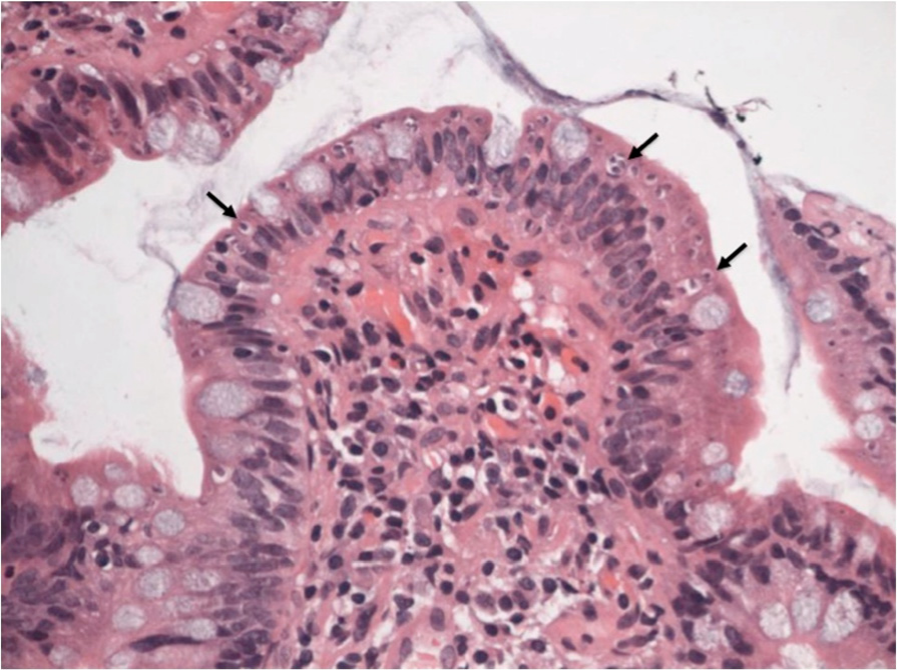
Histology of terminal ileum showing villus blunting and the presence of multiple intracellular protozoa (arrows) at the apical half of the enterocytes of the terminal ileum.

**Figure 2:**
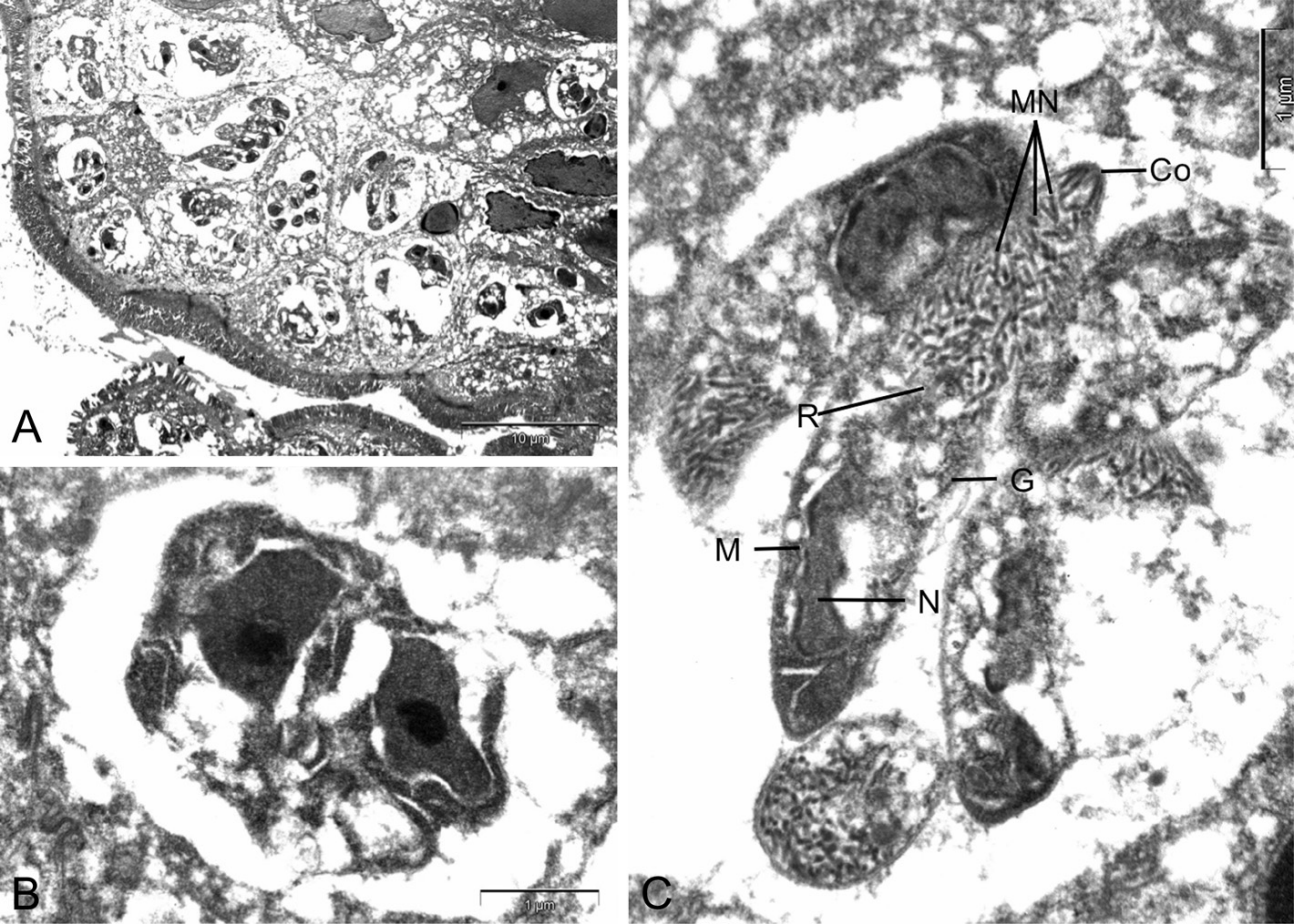
**Electron microscopy of the terminal ileum. Panel A**: The *Cyclospora* merozoites were surrounded by layers of thick parasitophorous vacuoles at the apical end of the enterocytes. **Panel B**: A binucleated schizont can be seen inside a parasitophorous vacuole. **Panel C**: *Cyclospora* merozoites showing typical features of an Apicomplexa organism: Co: Conoid with polar rings, G: Golgi body, MN: Micronemes, N: Nucleus, R: Rhoptry, M: Mitochondria.

**Figure 3:**
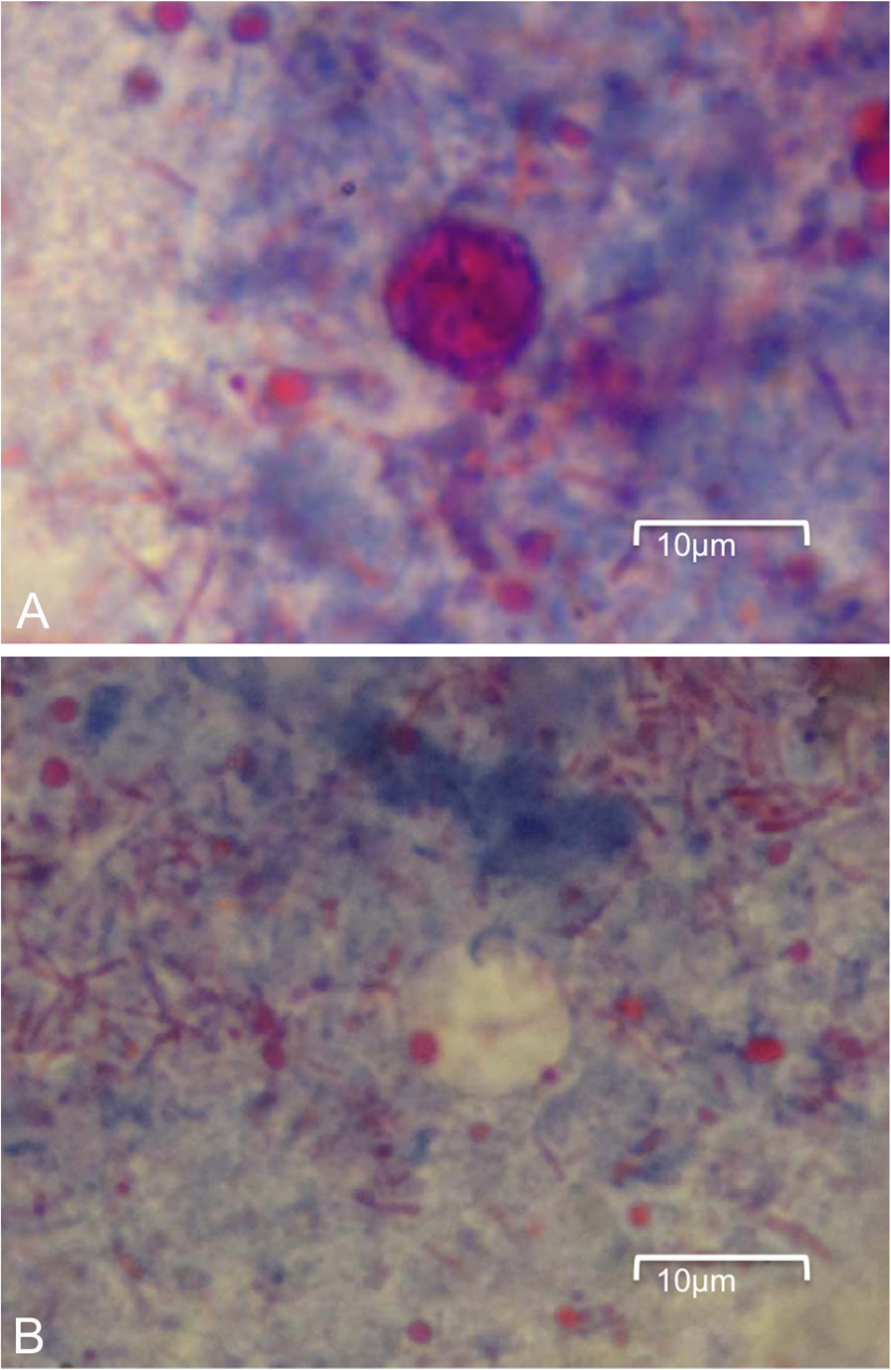
**Stool microscopy (modified Ziehl-Neelsen acid-fast stain). Panel A**: Circular *Cyclospora* oocyst of size about 9 microns. **Panel B**: An unstained *Cyclospora* oocyst, the “ghost” cell.

Microscopic examination and culture on repeated stool samples collected within 6 months’ time did not yield any bacteria, virus, or parasites initially. In view of the prolonged diarrhoea, an upper gastrointestinal endoscopy (UGIE) and a colonoscopy were performed. The UGIE only identified mild gastritis and the duodenum appeared normal. The colonoscopy showed only a small area of erythema over the descending colon with normal terminal ileum and other parts of colon. However, numerous intracellular protozoa could be identified at the apical half of the enterocytes of both the terminal ileum and the duodenum. Villus blunting with non-specific inflammatory cells infiltration of the terminal ileum was also noted (Figure 1). The electron microscopy of the parasite demonstrated a typical morphology of an Apicomplexa organism. The merozoites were surrounded by layers of thick parasitophorous vacuoles (Figure 2, Panel A). A binucleated schizont could be seen inside a parasitophorous vacuole (Figure 2, Panel B). Mature merozoites could also be identified (Figure 2, Panel C). Further stool samples using modified acid-fast staining showed typical *Cyclospora* oocyst (Figure 3, Panel A). An unstained oocyst, the “ghost” cell, could also be found (Figure 3, Panel B).

Further history revealed that her husband had multiple sexual partners in Vietnam. The lady was subsequently diagnosed to be HIV positive and the baseline CD4 count was only 98 cells/μL with HIV ribonucleic acid (RNA) of 420,000 copies/mL. She was initially treated with 2 weeks of ciprofloxacin 500 mg twice daily. However, her response was suboptimal. Trimethoprim/sulfamethoxazole (TMP-SMX) 960 mg twice daily was then introduced and her symptoms totally subsided after two weeks. Anti-retroviral therapy (ART) was also prescribed and her CD4 count went up to 153 cells/μL in half-year’s time. Her HIV RNA had become undetectable after 3 months of ART and she remained well. She also had weight gain of 5 kg after 6 months of ART.

## Discussion

Diagnosis of *Cyclospora* infection can be difficult because of its scarcity in developed countries, intracellular mode of life, small size of the parasite and its inability to take up routine microscopic stains. Difficulties in making a diagnosis of *Cyclospora* infection in our patient, especially in non-endemic areas, are illustrated. However, history of travelling to Vietnam with fresh fruit or vegetable exposure can be the clues, since the *Cyclospora* oocysts can be identified in vegetables, environmental water samples and patients suffering from diarrhoeal diseases in Vietnam [8-10]. In fact, the Vietnam coriander, which is commonly used in the Southeast Asian cooking, was found to be the most frequent vegetable contaminated with *Cyclospora* oocysts (11.6%) [8]. The symptoms of *Cyclospora* infection vary widely, depending on the immune status, age of the patient, and the infecting dose of the parasites. In endemic areas, the symptoms of infection are generally milder as a result of the presence of partial immunity. The vulnerable groups are usually the elderly and young children [11,12]. In non-immune individuals, the symptoms are more severe and usually come after a median incubation period of about 7 days. Typically they present with nausea, abdominal cramp, watery diarrhoea, low-grade fever and weight loss, lasting for weeks and even months [3,13]. Just as the case in our patient, the symptoms are more severe and prolonged in the HIV population [14,15]. Extra-intestinal forms of *Cyclospora* infection including biliary disease, acalculous cholecystitis or Reiter syndrome may occur, especially in the HIV population [15-17].

*Cyclospora* is an uncommon pathogen and many laboratories may not be experienced enough in making a diagnosis. Therefore routine stool examination for ova or cysts may not be adequate to pick up the *Cyclospora* oocysts. This was probably one of the reasons for the delayed diagnosis in our patient. Examining the fresh stool specimen for oocysts by using modified Ziehl-Neelsen acid-fast staining (MAFS) is helpful. However, the *Cyclospora* oocysts take up MAFS quite variably. The use of strong decolorizing agent in the MAFS may remove too much color in the *Cyclospora* oocysts, and it may then appear as unstained oocysts (ghost cells). In view of the small number of oocysts passed by the infected cases, the sensitivity for diagnosis may be enhanced by concentration and purification techniques [2,18]. Increasing the number of fecal samples for examination may further enhance the detection rate despite the absence of studies in this area. It is equally important to distinguish the oocysts of *Cyclospora* from those of *Cryptosporidium*, which is an even more common protozoa causing enteritis in HIV patients. In the past, the *Cyclospora* oocysts have been misdiagnosed as the “Big Crypto” since both oocysts are acid-fast [19]. However, the sizes of the oocysts can help to distinguish the two species. *Cyclospora* oocysts have a diameter of about 8–10 microns while those of *Cryptosporidium* measure about 5 microns [3,4]. As a result of the autofluorescence nature of the *Cyclospora* oocysts, the diagnosis can also be secured by examining the specimen under the fluorescence microscope. However, the sensitivity for microscopic detection may drop even with the concentration technique if the oocysts load is low, especially in patients with mild symptoms. Polymerase chain reaction may be helpful in this regard for its high sensitivity and specificity, though it is not widely available [3,20]. Unfortunately no serologic tests for *Cyclospora* are available so far. In highly suspected cases with negative fecal investigation, early enteroscopy with biopsy over small intestine may be imperative in making a diagnosis. Similar to our patient, there may be minimal or no sign of inflammation over the duodenum or terminal ileum under the endoscopic view [21,22]. However, typical histological findings of the small intestinal biopsy can be identified in our patient. They include blunting of the villi due to edema and infiltration by a mixture of inflammatory cells, vascular dilatation and congestion of the villous capillaries, and parasitophorous vacuoles containing sexual and asexual forms of *Cyclospora* protozoa [21,22]. Moreover, the parasites are usually present in the supranuclear area of the enterocytes and absent in the crypts [23].

*Cyclospora* infection is usually self-limiting in immunocompetent individuals, though relapse can occur. Seven to ten days of oral double strength TMP-SMX is the standard therapy [3,4,12]. The duration of treatment can be longer if symptoms persist. Many alternative medications have failed to demonstrate efficacies similar to that of TMP-SMX in eradicating the protozoa. A randomized trial in HIV patients has suggested that ciprofloxacin 500 mg twice daily for 7 days is an acceptable alternative to TMP-SMX, especially in patients with sulfa allergy [24]. However, the use of ciprofloxacin in our patient was not effective. Since recurrence can occur in up to 43% of *Cyclospora* infected HIV patients after effective treatment, secondary prophylaxis with double strength TMP-SMX three times weekly can be considered [14]. Nitazoxanide, a thiazolide agent with activities against many parasites, may also be considered in cases who have sulfa allergy or fail to respond to sulfa or ciprofloxacin [25,26].

## Conclusions

In summary, *Cyclospora* is an uncommon pathogen and may be difficult to be diagnosed. Meticulous history taking with special focus on travel and food intake is imperative. Microscopic examination of the concentrated fecal specimen using MAFS and /or immunofluorescence staining helps to identify the *Cyclospora* oocysts. Early enteroscopy with small bowel biopsy may be required in some cases and the typical histological findings can help to coin the diagnosis. TMP-SMX remains to be the standard therapy and quinolones appear to be less useful in some cases.

## Consent

Written informed consent was obtained from the patient for publication of this Case report and any accompanying images. A copy of the written consent is available for review by the Editor-in-Chief of this journal.

## Competing interests

The authors declare that they have no competing interests.

## Authors’ contributions

OTYT drafted and wrote the manuscript and provided therapy for the patient. RWCW performed histological examination, electron microscopy and contributed figures. JMCC, KYT and WSL were involved in the clinical guidance of the patient. BHSL performed the microbiological examinations and contributed figures. All authors read and approved the final manuscript.
